# Personalized medicine and Hispanic health: improving health outcomes and reducing health disparities – a National Heart, Lung, and Blood Institute workshop report

**DOI:** 10.1186/s12919-017-0079-4

**Published:** 2017-10-03

**Authors:** M. Larissa Avilés-Santa, John Heintzman, Nangel M. Lindberg, Rafael Guerrero-Preston, Kenneth Ramos, Ana L. Abraído-Lanza, Jonca Bull, Adolph Falcón, Mary Ann McBurnie, Ernest Moy, George Papanicolaou, Ileana L. Piña, Jennifer Popovic, Shakira F. Suglia, Miguel A. Vázquez

**Affiliations:** 1 Division of Cardiovascular Sciences, National Heart, Lung, and Blood Institute, 6701 Rockledge Drive, Room 10188, Bethesda, MD 20892-7936 USA; 2 Department of Family Medicine, Oregon Health and Science University, 318 SW Sam Jackson Park Rd, Portland, OR 97239 USA; 3 Kaiser Permanente Northwest Center for Health Research, 3800 N. Interstate Ave, Portland, OR 97227 USA; 4 Johns Hopkins University School of Medicine, 1550 Orleans Street, CRB2 Room 5M, Baltimore, MD 21231 USA; 5 University of Arizona Health Sciences, 1295 North Martin Avenue, PO Box 210202, Tucson, AZ 86721 USA; 6 Columbia University, Mailman School of Public Health, 722 West 168th Street, New York, NY 10032 USA; 7 Office of Minority Health, U.S. Food and Drug Administration, 10903 New Hampshire Avenue, Silver Spring, MD 20993 USA; 8 National Alliance for Hispanic Health, 1600 P St NW, Washington, DC 20009 USA; 9 National Center for Health Statistics, 3311 Toledo Road, Hyattsville, MD 20782 USA; 10 Albert Einstein College of Medicine, Montefiore Heart and Vascular Center, 111 East 210th Street, Bronx, NY 10467-2401 USA; 11 Program for Health Data and Standardized Methods, Center for Health Data Analytics | eHealth, Quality & Analytics Division, RTI International | 307 Waverley Oaks Road, Suite 101, Waltham, MA 02452 USA; 12 Rollins School of Public Health, Emory University, 1518 Clifton Rd Rm 4005, Atlanta, GA 30322 USA; 13 Department of Internal Medicine, University of Texas Southwestern Medical Center, 5323 Harry Hines Boulevard, Dallas, TX 75390-8856 USA

## Abstract

Persons of Hispanic/Latino descent may represent different ancestries, ethnic and cultural groups and countries of birth. In the U.S., the Hispanic/Latino population is projected to constitute 29% of the population by 2060. A personalized approach focusing on individual variability in genetics, environment, lifestyle and socioeconomic determinants of health may advance the understanding of some of the major factors contributing to the health disparities experienced by Hispanics/Latinos and other groups in the U.S., thus leading to new strategies that improve health care outcomes. However, there are major gaps in our current knowledge about how personalized medicine can shape health outcomes among Hispanics/Latinos and address the potential factors that may explain the observed differences within this heterogeneous group, and between this group and other U.S. demographic groups. For that purpose, the National Heart, Lung, and Blood Institute (NHLBI), in collaboration with the National Institute of Diabetes and Digestive and Kidney Diseases (NIDDK), and the Food and Drug Administration (FDA), held a workshop in which experts discussed (1) potential approaches to study medical treatments and health outcomes among Hispanics/Latinos and garner the necessary evidence to fill gaps of efficacy, effectiveness and safety of therapies for heart, lung, blood and sleep (HLBS) disorders and conditions--and their risk factors; (2) research opportunities related to personalized medicine to improve knowledge and develop effective interventions to reduce health disparities among Hispanics/Latinos in the U.S.; and (3) the incorporation of expanded sociocultural and socioeconomic data collection and genetic/genomic/epigenetic information of Hispanic/Latino patients into their clinical assessments, to account for individual variability in ancestry; physiology or disease risk; culture; environment; lifestyle; and socioeconomic determinants of health. The experts also provided recommendations on: sources of Hispanic/Latino health data and strategies to enhance its collection; policy; genetics, genomics and epigenetics research; and integrating Hispanic/Latino health research within clinical settings.

## Introduction

### Personalized/precision medicine and Hispanic/Latino health

Advances in biomedical research and clinical medicine have led to successful treatments for many diseases and significant improvements in health for many Americans. However, many innovative treatments have been developed to treat highly selected patient populations which may not be representative of clinically relevant populations. Despite efforts to advance clinical trial diversity, most trials remain representative of non-Hispanic White (NHWs) populations. Thus, large variations in disease course and response to therapy remain uncharacterized among culturally and racially diverse individuals with similar medical problems. Many of the health disparities documented in the United States are linked to access to care barriers and other socioeconomic determinants of health. But we do not have sufficient information to understand how the interaction between individual physiological/genetic/epigenetic variability, comorbidities, and other health determinants differentially affect members of most culturally and racially diverse communities in the United States, including Hispanic/Latino communities.

Precision medicine uses innovative biological, clinical and population data science tools to customize and personalize disease prevention, detection and treatment, leading to improvements in the effectiveness and quality of patient care [1]. A central goal of precision medicine is to shift disease management and prevention from generalized approaches to personalized care that takes into consideration individual variability in genes, environment and lifestyle [2]. Publicly available genomic and epigenomic tools and databases are fundamental tools for precision medicine efforts. Yet, most of the genomic data available has been collected on NHWs and ancestry related biases are not usually addressed in genetic and precision medicine studies [3, 4].

As of July 2015, the estimated Hispanic/Latino population in the U.S. was 56.6 million or 17.6% of the U.S. population [5]. It is projected that by 2060, the U.S. Hispanic/Latino population will be 119 million or 28.6% of the total population [5]. Persons of Hispanic/Latino background may represent different ancestries, ethnic and cultural groups and countries of birth, including the U.S. The term Hispanic often refers to persons who self-identify or trace their roots to Hispano American countries (former colonies of Spain), and to persons from Spain [6]. The term Latino may be interpreted differently in the U.S. and abroad. Latino refers to persons who self-identify or trace their roots to countries that are former colonies of Spain, Portugal and France (Latin Americans). Hispanic Americans, Hispano Americans or Latin Americans may also trace their roots to other European countries, the Middle East, Africa, and Asia. In the U.S., the term Latino often refers to Hispanics and Latin Americans, interchangeably. Place of living [7], heritage group [8] or immigrant generation [8] may influence an individual’s self-identification as Hispanic, Latino, both or neither. We use the terms *Hispanic* or *Hispanic/Latino* interchangeably, and both refer to persons who trace their roots to any Latin American culture.

Significant disparities in health between Hispanics/Latinos and other demographic groups in the U.S. have been amply documented. A personalized approach focusing on individual variability in genetics, environment, lifestyle and socio-economic determinants of health may advance understanding of some of the major factors contributing to the health disparities experienced by Latinos and other groups in the U.S., and lead to new strategies to improve health care outcomes. However, there are major gaps in our current knowledge about how personalized medicine can shape health outcomes among Hispanics/Latinos and address the potential factors that may explain the observed differences within this heterogeneous group and between this group and other U.S. demographic groups.

As part of a series of initiatives focused to make the benefits of precision medicine available to a larger portion of the population and to reduce health disparities among Hispanics/Latinos, the National Heart, Lung, and Blood Institute (NHLBI), the National Institute of Diabetes and Digestive and Kidney Diseases (NIDDK), and the Food and Drug Administration (FDA) planned and sponsored a workshop on July 6–7, 2016, at the main campus of the National Institutes of Health (NIH) in Bethesda, Maryland (USA): *Personalized Medicine and Hispanic Health: Contributions to Reducing Health Disparities.* The workshop’s main objectives were (1) identifying gaps in evidence of efficacy, effectiveness and safety of therapies for heart, lung, blood and sleep (HLBS) disorders and conditions--and their risk factors--accounting for the racial/ethnic and socioeconomic diversity among Hispanics/Latinos; (2) describing potential approaches to study medical treatments and health outcomes among Hispanics/Latinos and garner the necessary evidence to fill these gaps; and (3) defining research opportunities related to personalized medicine to improve knowledge and develop effective interventions to reduce health disparities among Hispanics/Latinos in the U.S. To fulfill these objectives, the sponsors invited medical and social science professionals, public and health policy experts, geneticists, epidemiologists, clinical investigators, and patient advocates to participate as speakers and panelists. The workshop was open to the public, and videocast live [9, 10].

The primary deliverable of the workshop, as detailed in each of the sections of our proceedings, was a set of specific and actionable recommendations to address pervasive health disparities among Hispanics/Latinos living in the U.S., and advance research in Hispanic/Latino health with personalized medicine as the ultimate goal. These recommendations represent the consensus opinions expressed by workshop presenters and ratified by contributing authors during the review of the manuscript. Recommendations were summarized for publication with the goal of guiding future efforts among workshop participants and to share specific action items with key stakeholders interested in reducing health disparities and improving health outcomes among Hispanics/Latinos as the evolution of precision/personalized medicine continues to unfold. Implementation of these recommendations are expected to help national initiatives improve efficacy and safety by properly accounting for racial/ethnic and socioeconomic diversity among Hispanics/Latinos, increase accessibility to medical treatments and improved health outcomes and narrow gaps in knowledge and translation of findings for personalizing disease prevention and treatment for Hispanic/Latino patients. The final version of the manuscript was reviewed and approved by National Institutes of Health program officials.

We begin our summary of the workshop with an overview of current research-based knowledge of cardiovascular and pulmonary diseases in Hispanic/Latino populations in the U.S. and participation of Hispanics/Latinos in clinical research discussed during the workshop. We will then highlight recommendations from the workshop on: sources of Hispanic/Latino health data and strategies to enhance its collection (especially accounting for variability in ancestry, disease risk, culture, environmental factors, lifestyle and socioeconomic determinants of health); policy, genetics, genomics and epigenetics research; and integrating Hispanic/Latino health research within clinical settings. We prefer the term *personalized medicine* over precision medicine because the person is at the center of individualized preventive and medical care.

## Hispanic/Latino Health Research landscape and opportunities

### Cardiovascular and pulmonary diseases in Hispanics/Latinos in the U.S

Despite being part the fabric of the U.S. population since the country’s inception, the inclusion of Hispanic/Latino individuals or populations in research studies has been minimal and regionalized. The first national cross-sectional study on Hispanic/Latino health, the Hispanic Health and Nutrition Examination Survey (Hispanic HANES) took place in 1982–1984 [11], and included 16,000 individuals of Mexican, Cuban and Puerto Rican origin. The inclusion of Hispanics in the National Health and Nutrition Examination Survey (NHANES) has primarily represented those of Mexican or Mexican American ancestry [12]. Other observational cross-sectional and longitudinal studies have focused on a specific Hispanic/Latino heritage group [13–18]. There has been a longstanding assumption that Hispanics/Latinos are an ancestrally, culturally and socioeconomically homogeneous group, and the number of studies exploring their differences and similarities (which would enrich the foundational knowledge towards precision/personalized medicine) has been limited [11, 19]. Therefore, the Hispanic Community Health Study/Study of Latinos (HCHS/SOL) has become the most comprehensive study on the health of the contemporary Hispanic/Latino population of the U.S. [20]. In this study, 16,415 individuals who self-identified with Central American, Cuban, Dominican, Mexican, Puerto Rican and South American heritage groups were examined in four U.S. cities, and are followed annually. The national vital statistics report denotes cancer is the leading cause of death among Hispanics/Latinos [21], but cardiovascular disease (CVD) is the second most common cause of death in this population [21]. This study seeks to understand whether there are differences or similarities in cardiovascular and pulmonary health profiles among the different heritage groups at baseline, and if the relationship between the baseline health profile and future health outcomes would differ by heritage group.

The baseline examination, which took place from 2008 to 2011, revealed both similarities and differences in the prevalence of traditional cardiovascular risk factors (obesity, hypertension, diabetes mellitus, dyslipidemia and tobacco use) and other disorders among heritage groups [22]. Diabetes mellitus was more prevalent among Hispanics/Latinos of Dominican, Mexican and Puerto Rican heritage, and less prevalent among those of South American heritage [23]. Differences and similarities in hypertension [24], nutritional intake [25], dietary quality and its relationship to cardiovascular risk factors [26], metabolic syndrome [27], dyslipidemia [28], smoking [29], and sleep disordered breathing [30] were also observed among different heritage groups.

Bronchial asthma was significantly more prevalent among those of Puerto Rican heritage, and lowest among those of Mexican heritage [31]. Chronic obstructive pulmonary disease (COPD) was significantly higher among Hispanics/Latinos of Puerto Rican and Cuban heritage compared to Hispanics/Latinos of other studied heritage groups [31]. In some of these HCHS/SOL analyses the authors described associations between health measurements and socioeconomic indicators, place of birth (U.S.-born versus foreign-born), years living in the U.S., preferred language, and other indicators of acculturation [22, 23, 27–29]. However, those associations have not been consistently positive for every CV risk factor. In addition, genetic studies of the population’s principal components [32] and local ancestry [33] revealed substantial genetic differentiation within and among the six heritage groups. It is not completely known whether differences or similarities in genetic ancestry explain the observed phenotypic similarities or differences among Hispanic/Latino heritage groups.

These findings indicate that to reach a deeper understanding of the variations in health-related factors among Hispanics/Latinos in the U.S. and abroad, additional factors ought to be systematically considered. Personal and family medical history, socioeconomic and sociocultural factors throughout a person’s life course and across generations, reasons for immigration or migration, history of exposures (e.g. infections, nutrition), education, health beliefs, and practices prior to and after migrating to the U.S. all may potentially shed light on individual or group risk for or protection from specific diseases. In addition, years living in the U.S. and stratification by generation in the U.S. provide context for assessing risk for diseases endemic in the country of origin (e.g., Chagas’ disease [34], gastric cancer [35]), versus others more common in the U.S.

In addition to the factors described above, health outcomes for any population may be significantly influenced by access to and quality of healthcare. Since 2003, the annual National Healthcare Quality and Disparities Reports (QDR), generated by the Agency for Healthcare Research and Quality, have shown that Hispanics receive poorer quality health care [36] than NHWs for about 40% of the measures tracked.

Care for cardiovascular and respiratory diseases mirror these overall patterns as summarized in the 2014 QDR Chartbook on Health Care for Hispanics [37], with Hispanics/Latinos frequently receiving poorer quality care for hypertension, heart attack, heart failure, and asthma. For example, compared with NHWs, non-Mexican Hispanic adults are 11% less likely to be screened for hypertension and Mexican Americans with hypertension are 35% less likely to achieve blood pressure control. Hispanic adults have higher hospitalization rates than NHW adults for hypertension, angina, and congestive heart failure. Hispanic men with asthma are 40% less likely than NHW men to take daily medicine to prevent asthma attacks. Compared with NHW children, Hispanic children have 60% more emergency department visits for asthma and 70% more hospitalizations for asthma. In California, Hispanics, especially Spanish speakers, are more likely to be hospitalized in hospitals with low communication scores than NHWs; non-English speakers as a whole are more likely than English speakers to be readmitted following discharge for heart attack or heart failure. While disparities have narrowed for hospital care, disparities remain between Hispanics and NHWs in outpatient care settings and in cardiovascular and respiratory care outcomes.

### Participation of Hispanics/Latinos in clinical research

Clinical trials are an important potential source of information concerning the presence of cardiovascular, pulmonary, blood and sleep diseases/disorders and their risk factors among Hispanics/Latinos. While some of these trials have been rather large [38–40], and adequate representation of Hispanics/Latinos would have been expected, their number has been small. For instance, the percent of Hispanics/Latinos in large clinical trials such as the Antihypertensive and Lipid-Lowering Treatment to Prevent Heart Attack Trial (ALLHAT) [38], Systolic Pressure Intervention Trial (SPRINT) [39], and Action to Control Cardiovascular Risk in Diabetes (ACCORD) [40] ranged between 7 and 12.5. Similarly, the National Emphysema Treatment Trial [41] of lung volume reduction surgery included 1218 patients, of which 99% were deemed as NHW or African American. These low numbers of Hispanic participants may limit the ability to complete heritage subgroup analyses of treatment effects and outcomes.

In 2012, as part of the FDA Safety and Innovation Act (FDASIA) [42], Congress required the FDA to develop (a) a report on the extent to which demographic data was being collected, analyzed and shared in labeling new safety information over the product life cycle; and (b) an action plan based on the report and stakeholder input. Since 1998, under what is known as the “demographic rule”, the FDA has required that sponsors for pharmaceutical and biologic products submit data that identifies sex, race and age [43]. However, concerns were raised by stakeholders concerned about low participation in clinical research by minority groups, and a notable lack of consistent processes to collect data on Hispanic/Latino participation [43, 44]. Consequently, the FDA has issued a revised guidance for reporting of race and ethnicity in clinical trials for the industry and FDA staff [45].

It is anticipated that identification of these issues and the FDA’s resulting reporting regulations will lead to strategies that increase participation by Hispanics/Latinos (as well as other minority groups). Barriers to recruitment of and participation by Hispanics/Latinos in clinical trials and personalized medicine research studies must be addressed if we are to develop a personalized medicine approach to Hispanic/Latino health.

### Collecting Hispanic/Latino health data

To apply a personalized medicine approach to the diagnosis, treatment and prevention of HLBS and other chronic diseases in Hispanics/Latinos, research is needed in both efficacy and effectiveness of diagnostic and treatment modalities--especially within the context of comorbidities, and factors mentioned above that contribute to the diversity of Hispanics/Latinos. Health care systems, biobanks, registries and claims-based administrative databases hold tremendous potential for a personalized medicine-based approach to the study of Hispanic/Latino health.

- *Health Systems:* The use of electronic health records (EHRs) has increased significantly during the last two decades. EHRs have the potential to contribute to knowledge regarding patient-level clinical data, including the assessment of continuity of care and comparative effectiveness of treatments; monitoring the impact of new interventions [46]; eliminating unnecessary, inefficient, or ineffective activities; and addressing population-level questions, such as identification of disparities related to prevalence and treatment of health conditions [47–52].

Health systems, particularly those with centralized and searchable EHRs, may have extensive data on Hispanic/Latino patients, including information on sociodemographic factors, longitudinal service utilization, diagnoses, biomarker data, and insurance coverage (including the uninsured) [53–56]. These types of data have been used in a variety of published analyses on healthcare access, health insurance, and preventive service utilization [57–59].

Challenges with EHR-based health systems data include the heterogeneity in data format, completeness and quality, data collection and management methods, and a lack of interoperability across multiple sites, including ambulatory and hospital environments. Some EHR-based data sources may reflect only ambulatory or in-patient health data. Hispanic/Latino subgroup data are rarely collected uniformly or in significant volume to merit comparisons.

- *Biobanks:* Store-housed biological samples may provide a large volume of genetic data for prospective evaluation of the genetic and epigenetic contribution to health in Hispanics, and are the focus of recent federal initiatives [60]. Challenges with the use of biobanks for research studies include ensuring a representative sample of individuals from which samples are gathered, linking genetic data to phenotype and environmental data, a limited number of conditions for which genetic information has been shown to be predictive of risk or outcome, ethical issues such as informed consent, and costs in reporting actionable genetic results to research study participants.
- *Registries:* Patient and disease registries have also been used to describe populations, including Hispanics, and to track the epidemiology of certain conditions and risk factors [61–63]. Registries often have detailed biological, demographic, and health care information, and cover wide geographic areas and demographic groups [64]. However, registries are limited to certain disease processes, exclude those without specified conditions, and depend on geographic location, initial access to care and subsequent patient involvement to collect data over time. They also lack denominator data; that is, data on the population from which the registry patients were drawn. These features may limit certain at-risk populations from entry into registries [65].
- *Claims-based databases:* Health insurance claims (or administrative) data allow payers to reimburse health care providers for services rendered. This central billing role also drives the strengths and weaknesses of the use of claims datasets for health research in Hispanic populations. Strengths include the capture of the breadth of an individual’s or population’s billable, medically attended care over a defined period of observation (health insurance enrollment), regardless of specific provider and facility. Claims are also a cost-effective data source for research-related studies, since they are readily available and do not require any primary data collection.

Limitations of claims-based systems include variation in the quality and completeness of individual data elements on a health care claim form, particularly the lack of reliable race/ethnicity data, and missing information related to out-of-pocket and over-the-counter services. In claims-based systems, information is limited to insured populations, and lacks clinical detail regarding services for which a specific bill is not rendered. Finally, there may be a specific time lag between the provision/documentation of healthcare events and services, and their appearances in the data warehouse. Claims- and EHR-based systems provide structured data (not free-text clinical notes) captured via well-established coding systems, such as International Classification of Diseases, Ninth Revision, Clinical Modification (ICD-9-CM) (diagnosis and procedure codes used for diagnostic, billing and reporting purposes and mortality coding) [66]; ICD-10 CM (most current version of the ICD) [67]; Current Procedural Terminology (CPT) Codes (determine amount of reimbursement for services provided) [68, 69]; and National Drug Codes (NDC) Directory (universal product identifier of human drugs in the United States [70]. However, improvements in interoperability are needed. Natural language processing methods might be used to gain additional knowledge from EHR notes fields [71–73].

### Expanding traditional clinical data collection

In the context of personalized medicine, the study of human genetic variation is particularly important because genetic variation may account for differences in health outcomes among people from different ethnic or racial groups. This is particularly true for Hispanics/Latinos; it has been demonstrated that despite a less favorable CVD risk factor profile than NHWs and non-Hispanic Blacks, Hispanics exhibit lower cardiovascular mortality, a pattern referred to as the Hispanic Paradox [74, 75]. Based on this and similar paradoxical relationships there has been resurgence of interest in the study of genetic admixture and its impact on the amalgam of races and ethnicities that make up the U.S. population.

In addition to genetic susceptibility, extensive work has documented a relation between social and cultural factors as they impact cardiovascular and pulmonary health. However, research gaps exist on the mechanisms by which these social determinants impact health. Socioeconomic and cultural factors may modulate health and disease via biological mechanisms linked to epigenomic alterations [76, 77], microbiome changes [78, 79], and higher allostatic load [80]. For example, stressors may be experienced across the life course, and research suggests that early childhood exposure to adversity is associated with negative health outcomes in both childhood and adulthood [81, 82]. Epigenomic-mediated stress responses may be one of the molecular mechanisms by which the social environment becomes biology.

To maximize efficacy, a personalized medicine approach might take into account sociocultural and structural contexts that promote or present barriers to health. For example, referrals for medical treatments and regimens to improve cardiovascular health (e.g., medications for hypertension, plus a physical activity regimen) must consider the built environment (lack of safe walking trails or exercise facilities) that hinder the ability to follow the prescribed regime. Such lifestyle approaches may hold the greatest promise in tailoring medicine, genetics, and individual biology to the environment in which individuals live [83].

## Discussion- recommendations

The final workshop discussion was dedicated to potential strategies for effectively implementing research within clinical settings, and for engaging stakeholders in this process. The following are the key recommendations resulting from this discussion.

### Improve data collection strategies through existing networks of health care systems and clinics

While the implementation of current medical guidelines for prevention and care should continue, relevant data to help us understand the effectiveness and safety profile of therapies for HBLS diseases and their interaction with other factors across Hispanic/Latino heritage groups needs to be simultaneously assessed. These data would be best collected through a dedicated network of health care systems and clinics, in concert with other databases described above. Such a dedicated network could be based on existing platforms and designed to leverage the expertise of regional health information organizations. And considering advantages and disadvantages of these databases previously described (e.g., health systems, biobanks, registries, claims-based databases), potential opportunities to enhance and expand these should be explored.

- In addition to clinical and demographic data (e.g., existing medical conditions, hospitalizations, medications), datasets should be enriched with expanded information on Hispanic/Latino identifiers, place of birth, travel and migration history, and expanded socioeconomic assessments; effectiveness and interaction of pharmacological and non-pharmacological treatments; genetic/genomic and molecular data; drug/surgery adverse events; comorbidities; use of over-the-counter drugs and dietary supplements, and traditional herbal and non-herbal remedies; and changes in nutrition and physical activity.
- Standardized data dictionaries focused on Hispanic/Latino health are essential and should be created.
- Appropriate expertise must be integrated into data networks to link datasets and systems efficiently and effectively. This kind of linking (for instance, claims and EHR databases) is expensive and time consuming, but can be very valuable for understanding personalized medicine. The interoperability of EHR systems could be enhanced by policies, such as the required collection of standardized key data elements (e.g., more granular ethnicity, country of birth, parents’ country of birth) and data management processes (e.g., patient matching procedures) to improve the completeness and quality of EHR data. Examples of such policies include Meaningful Use Core Measure on Smoking Status [84], HRSA’s collection of sexual orientation/gender identity data [85], and ONC’s Patient Matching Initiative [86, 87]. Using and expanding existing models of already integrated systems and leveraging the expertise of established networks could save time. As in any health network, a methodology to search for and handle missing data needs to be developed and validated.

### Require inclusion of race/ethnicity data in all health-related datasets

A minimal set of identifiers of Hispanic/Latino heritage/identity should be incorporated into data collection platforms. Race/ethnicity self-identification is preferable over identification assigned by administrative staff, researchers or health care providers, since it is likely to be more accurate. Self-identification should be expanded beyond the blanket terms “Hispanic” or “Latino” to include more granular information on nationality, cultural ancestry and/or heritage [88]. This minimal set of identifiers should include standard definitions, which could be incorporated into the EHR.

### Strengthen guidance to researchers by rewarding inclusive study designs

Besides the current policy on inclusion of women and minorities as subjects in biomedical research [89], the processes for reviewing and awarding of grants could place greater value on (1) studies demonstrating power of the subject sample by sex, race, and ethnicity, when appropriate; (2) a plan to report findings by sex, race, and ethnicity, and eventually publish the findings; (3) innovative models of inclusion and academic-community partnerships, like community-based participatory research, when appropriate; and (4) applications proposing sites for multicenter grants that represent the diversity of the U.S. population, including the diverse Hispanic/Latino heritage groups. Furthermore, it is critical that review boards reflect inclusive excellence. These steps will help improve the current status quo whereby Hispanics/Latinos represent 17.4% of the U.S. general population, but 7.2% of biomedical doctoral degrees, and only 3.4% of NIH-funded principalinvestigators [90].

### Integrate genetic and epigenomic data into the electronic medical record

Advancing our current understanding of Hispanic/Latino health will require the use of genetic and epigenomic data to more precisely define the taxonomy of disease processes and to optimize therapies, while minimizing adverse reactions. Such interventions have proven extremely valuable in the management of several diseases including cardiomyopathies [91, 92] and diseases of the lung [93]. To ensure that precision medicine pipelines include Hispanic/Latino populations, molecular research efforts must be set in motion to identify: 1) genetic and epigenomic biomarkers associated to causal or risk factors; 2) underlying genetic and epigenetic alterations that lead to manifest disease, and 3) genetic and epigenomic markers relevant to prognosis and prevention.

To achieve these goals, it is imperative to increase genomic/epigenomic research Hispanic/Latino populations. For instance, Popejoy and Fullerton reported that in 20 years the representation of Hispanics/Latinos in genome-wide association studies rose from 0.06% to 0.54% [94]. The participation of the HCHS/SOL in the Trans-Omics for Precision Medicine (TOPMed) Program is helping to change these numbers, since TOPMed aims at collecting and assembling -omics data across diverse populations including those traditionally underrepresented in research [95]. Furthermore, analyses should focus on novel findings as opposed to confirmation of variants or genes from non-Hispanic populations and be devoid of biases that arise when cross-population analyses are prioritized towards non-Hispanic populations. Resources need to be developed that explore and make available both population specific variants and methods that incorporate admixed populations, as well as ancestral variation. Importantly, however, genetic and epigenomic data that define the biological contributions of admixture should be combined with the study of the epigenome at different life stages. Personalized medicine will be driven by personalized maps of epigenomic alterations of disease risk and causality associated to health behavior, neighborhood effects, allostatic load, the microbiome and other health determinants in Hispanic/Latino populations.

### Prioritize research on Hispanic/Latino populations not currently represented in national studies or the *All of Us* initiative

Originating from over 20 countries, Hispanic/Latino heritage groups in the U.S. have diverse sociodemographic and health profiles [96, 97]. Although there is growing recognition of differing health patterns and profiles [96, 97], data on health or health behaviors are still scarce for specific heritage groups and for geographic locations in which Hispanic/Latino communities are emerging or have been recently established.

Many Hispanic/Latino health research studies have been based on samples in California and the Southwest, followed by some locations in the Midwest and the Northeast. However, very few draw from other geographic locations with a significant percent of Hispanics/Latinos (e.g. the Washington DC area) or with emerging Hispanic/Latino communities [98]. Salvadorans and Dominicans constitute the fourth and fifth largest Hispanic/Latino heritage groups in the U.S, respectively. However, they are seldom represented in research studies or national surveys. Puerto Rico, a U.S. territory, is included in the Behavioral Risk Factor Surveillance System (BRFSS) [99], but not in National Health and Nutrition Examination Survey (NHANES) [100], the Multi-Ethnic Study of Atherosclerosis (MESA) [101] or the HCHS/SOL [20].

To assure progress in promoting the best health outcomes for all, research programs must include meaningful participation from all Hispanic/Latino heritage groups represented in the U.S. population.

### Integrate Hispanic/Latino health-related research within clinical settings

More research in Hispanic/Latino health should be performed in community clinical settings rather than academic settings for the following reasons.

- Research in community clinical settings offers the possibility of collecting new data and accessing existing relevant data, addressing patient-centered concerns, and improving patient care outcomes.
  Clinical settings provide the ability to identify potential research participants with specific exposures or diagnoses through EHR, ascertain health outcomes, validate diagnosis codes, estimate disease or risk factor incidence rates, and identify persons with new disease onset [51, 102]. In addition, having access to health care providers in clinical settings may illuminate barriers and facilitators to recommended care guidelines. Quasi-experimental designs and cluster-randomized studies provide pragmatic research approaches that mitigate the research burden on clinic workflow [103].
- Results of research based in clinical settings can be more generalizable. The patients served in community clinical settings or health systems experience a broad range of healthcare environments, social determinants of health, and complex co-morbidities [51]. The results of research studies in these settings would be more representative of those communities than results of research carried out in academic settings, which tend to be highly selective with respect to patients, providers and healthcare environments [49–52].

Research integrated into clinical settings also presents unique and difficult challenges. A recent survey of PCORnet awardees and stakeholders identified those challenges [104] which included: excessive burden on clinic staff and clinic resources, researchers’ lack of knowledge about some aspects of the health care enterprise (i.e., customer service, resource utilization), clashing of differing professional cultures, and low patient engagement.

Inadequate access to health care is a barrier to the benefits of new discovery and precision medicine; about 20% of Hispanics remain uninsured compared to 10% of NHWs [105]. The inclusion of safety-net-based health-care settings/data resources will capture at least some of these patients and should be mandatory for research involving Hispanic/Latino populations.

Finally, approaches to enhance participation of Hispanics/Latinos in research should consider patients’ educational attainment, employment-related (e.g., work shift, lost wages), transportation, preferred mode of communication (in-person versus phone call versus mail), preferred language, past experience with medical or government systems, cultural beliefs and personal expectations about health maintenance and disease treatment and prevention, among others.

### Engage stakeholders at multiple levels of the research enterprise

The collaborative efforts of multiple system stakeholders working together toward a common goal could significantly reduce barriers to successful conduct of biomedical research involving Hispanic populations [106]. Team science and inclusion of non-scientific experts in the research design should be promoted. Teams should include clinicians, experts in clinical trials, social/psychosocial experts, genetics/genomics experts, recruitment experts, case managers, administrative personnel, information technology analysts, interpreters, patient advocates, patients, community members, educators, and students.

Optimal stakeholder engagement is fostered by a bidirectional approach (both top-down and bottom-up) in communication in all stages of research, from planning to implementation. Participation of administrators and front-line clinic staff in the planning stages of a research project is likely to result in a more seamless transition between clinical and research tasks that staff members are often involved in. This transition will also increase staff members’ sense of ownership of the research project, which is in turn may increase the likelihood of project success.

Clinicians of Hispanic/Latino origin who are associated with academic medical centers or community clinical centers may play a key role in educating Hispanic/Latino patients about clinical trials and how they could contribute towards the understanding of their own health and their families’ health. Also, investigators in academic institutions may invite physicians in the community to become points of referral of Hispanic/Latino patients, and, in turn, become an integral part of the research team. Finally, the creation of a national Hispanic/Latino Patient Advisory Panel could identify, engage in and advocate for resources and policies that would support enhanced research among Hispanic populations.

### Increase and enhance Hispanic/Latino scientific workforce

Increasing the participation of Hispanic/Latino individuals in all aspects of the health-care workforce, from healthcare to scientific research, is essential to provide culturally competent care to the growing U.S. Hispanic/Latino population [107]. It is imperative to increase Hispanic/Latino access to educational opportunities, and efforts to identify, recruit, and retain talented Hispanic/Latino students in the STEM and biomedical disciplines pipeline starting at the elementary school level, and to prospectively plan recruitment of talented Hispanic/Latino scientists and promotion of Hispanic/Latino faculty to positions of leadership in academia and funding agencies. Hispanic/Latino researchers or health care providers may enhance awareness of ethnicity-based differences in disease prevalence or symptomatology, which could reduce health disparities and improve treatment outcomes in this population [108].

## Conclusions

We know that diversity in our scientific enterprise is vital to improving the health care of our nation. Recent research has confirmed what has long been suspected: there are major differences in the safety and effectiveness of many treatments, with a key variable being the gender, race and ethnicity of the individual. The only way we can make progress in building a strong foundation for personalized medicine is to meaningfully include all Hispanic/Latino heritage groups represented in the U.S. population in health-related research projects. We hope that the recommendations of the *NIH Workshop on Precision Medicine and Hispanic Health* will move the nation forward in securing the best health outcomes for all.
